# Lectures on Diseases of the Chest

**Published:** 1845-06

**Authors:** J. R. Buck

**Affiliations:** Lecturer on the Theory and Practice of Medicine in the Louisville Summer School of Medicine; Louisville


Art. II.—*Lectures on Diseases of the Chest.* By J. R. Buck, M.D., Lecturer on the Theory and Practice of Medicine in the Louisville Summer School of Medicine.

### LECTURE THIRD.

*Phthisis.*—By Louis and many other distinguished pathologists, tubercular matter in the lungs has been regarded as the cause and characteristic of this disease; and doubtless it is the cause of that group of symptoms, cough, pain in the chest, expectoration, hectic fever, &c., to which the term phthisis is popularly applied; but we must extend our inquiries further, and ascertain the cause of this deposit—the *causa causata.*

Tubercular matter does not exist in the lungs, or in any other cavity of the body as a natural condition, nor is it the product of a healthy action; it must therefore be preceded by morbid action, which morbid action, of whatever nature it may be, is the primary disease, the cause, while tubercular matter is the secondary disease, the effect of this cause. And as tubercular matter is found in almost every organ of the body this action must be general, pervading at least all the organs in which this deposit is formed. There is no propriety in regarding tubercles rather than hectic fever as the disease; strictly speaking, they are both diseases, but they are secondary to some primary disease—that is, effects or symptoms: regarding them therefore in this light, we are led to inquire, 1st, what is the nature of that primary morbid action of which these are the symptoms; and 2dly, are there any means by which we can detect this morbid action previous to the development of these symptoms? In the present state of our knowledge it is not possible to give an entirely satisfactory answer to the first of these questions. By many distinguished pathologists, and the current of popular opinion at the present day seems to be rapidly tending that way, this primary disease is regarded as inflammation, while by others equally distinguished, a morbid action directly opposed to inflammation, is regarded as the true cause of the tubercular deposit. What is the evidence upon which the opinion of its inflammatory origin rests?

1st. It is contended that tubercular matter is frequently found co-existing with well known products of inflammation, congestion, coagulable lymph, pus, &c.

2d. That tubercular matter is organized, containing blood-vessels susceptible of being injected, and softening from the centre.

The mere co-existence of tubercular deposits and inflammation certainly does not prove the dependence of one upon the other, and if it does, it no more follows that inflammation is the cause of tubercle, than that tubercle is the cause of inflammation. If tubercle was the product of inflammation, should we not find it oftenest when inflammation is most frequent, and should not those organs or parts of organs least liable to inflammation present a corresponding exemption from this deposit? Is this in accordance with the facts? In what organ is this deposit most frequently found? The lungs. Are the lungs oftener inflamed than any other organ of the body? They are not; gastritis and enteritis are far more common than any inflammation in the chest, and yet tubercular matter is rarely found in these localities, compared with its great frequency in the lungs. And in what part of the lungs is inflammation most frequently seated? The middle and lower lobes; while tubercle is almost uniformly seated in the apices; so constant is this, that the diagnosis of phthisis from chronic pneumonia often turns upon the location of certain phenomena; thus dullness on percussion, rude or bronchial respiration, at the base of the chest would indicate pneumonia or some other inflammatory disease, because this is the point at which inflammation is usually seated, while the same signs occurring at the summit of the lungs would indicate the existence of tubercular matter, this being the point where it is first deposited.

Miliary granules do not constitute the first stage of tubercle, but sometimes exist for a very long period without manifesting the slightest tendency to pass into yellow or genuine tubercle. A Frenchman was brought into Blockley hospital with chronic peritonitis; he died dropsical several months after his entrance. The centre peritoneal membrane was studded with miliary granules, they had doubtless existed there from the attack of peritonitis, and yet there were no tubercles, and apparently no tendency, in any one of them to undergo any such change. A similar case occurred in this city, but a few months since, and some of the present class witnessed the examination of the body.

But it is contended that these granules sometimes occur in the lungs, and that they then follow the same order both in their development and in the changes which they undergo, that tubercular matter does. This matter exciting active inflammation in the adjacent structure of the lungs, coagulating lymph is thrown out, forming small indurated, semi-organized points—which constitute the miliary granules, and of course are developed as the tubercles are deposited and excite inflammation. Thus, granules become points of irritation, attracting to them more blood than exists in the surrounding healthy tissues, and therefore more liable to be the seats of deposits or secretions from the blood. Once existing here, it undergoes the same changes, and in the same order that it does when deposited in any other portion of the lung, and is thus mistaken for genuine tubercle passing through its different stages. Again, the granules are found in organs, the uterus, &c., in which tubercle is never seen.

The organization of tubercles rests upon the facts that blood-vessels have been injected into them, and that they begin to soften from the centre. If blood-vessels exist in the tubercular mass, as an essential constituent, then there can be no question of its being an organized substance. In some few instances, by a very delicate injection, one or more vessels have been seen to penetrate into or entirely through a tubercle. Now if the vessels were essential constituents of tubercle would they not exist, and would they not be found almost uniformly in tubercles of the same size; and in those of double the size would not the vessels be either twice as large or twice as numerous? In every organ of the body, in every living animal, the size and number of the blood-vessels bear an exact ratio to the size of the organ supplied by them. But in tubercle no such proportion is observed; in a very small tubercle a vessel may occasionally be seen, while in others of the same or double the size no trace of a blood-vessel is discoverable. How then are these vessels formed? The tubercular matter is a secretion from the blood; it is thrown out so as to surround one or more blood-vessels—this matter may either partially or entirely obliterate the vessel by compression or it may have no effect upon it, being so moulded around the vessel as not to compress it at any point; in large tubercles when softening has taken place, when they have been converted into purulent matter, these vessels are still seen passing through the mass with the circulation uninterrupted; and even after this matter has been expectorated leaving an empty cavern, bands formed by blood-vessels in which the circulation is natural, are frequently seen passing across them. The rupture of these vessels is the cause of sudden death occurring in the advanced stages of phthisis. The blood-vessels then, met with in tubercle, properly belong to the healthy pulmonary structure, and have no ther connexion with tubercular matter, than that they pass through it, just as an artery or bronchial tube is sure to pass through a purulent mass, caused by suppuration of the surrounding tissue. Tubercles sometimes soften from the centre.

When this deposit first occurs it is in a fluid state; this fluid is gradually removed by absorption, the internal portion remaining till the last, so that when the surrounding tissue secretes pus, and this dissolves the matter, an excavation is formed in the centre, as though the softening had commenced at that point. In another case the softening does commence in the centre. The tubercle in its deposition encloses a small portion of the pulmonary structure which undergoes decomposition; is converted into purulent matter and dissolves the mass. But in a large majority of cases the softening commences at the circumference, the surrounding tissue throws out pus which insinuates itself between the particles of tubercle, and breaks it down dissolved in the pus. Such are the most prominent arguments for the inflammatory origin of tubercle. I have shown that inflammation most frequently occurred where tubercular matter is rarely formed—in the lower lobe of the lungs; and that tubercular matter is almost uniformly first deposited at the apices of the lungs, where inflammation rarely exists. The observations of that most accurate of all observers, M. Louis, upon many hundred subjects, show that inflammatory diseases, pneumonia, pleurisy, bronchitis, &c., &c., do not in the slightest manner predispose to phthisis when occurring in persons previously healthy, and that those occupations which produce bronchitis such as needle-pointing, fork grinding, &c., have no influence in the development of tubercle. It has also been shown by Andral that in inflammation, fibrin of the blood is increased, while in the earliest stage of tubercular deposit this element is not above the healthy standard; and in the tubercular diathesis before the matter secreted, the fibrin is diminished.

The tubercular diathesis is characterized by a weak, relaxed condition of the muscular system, pallor or dusky hue of the skin, transparency of the eyes, &c., while the inflammatory diathesis is characterized by a florid complexion, with great muscular energy. The ordinary products of inflammation are serum, lymph, and pus, and when any other morbid products are seen, whether consisting with these or not, it must be referred to some other morbid action.

What then is the nature of this primary disease? The only morbid condition constantly found is an altered state of the blood, and hence to this alteration must be referred the deposit of tubercular matter in the lungs.

The blood is deficient in fibrin; the serous or watery portion is increased, and the coloring matter and globules diminished. Other alterations of this fluid may also exist, but they have not as yet been detected.

The mucous membranes seem also to be diseased, perhaps as a consequence of the diseased condition of the blood. The lungs with the exception of the mucous lining of the bronchial tubes are no more diseased than any other organ of the body.

It is not possible to pronounce with entire certainty as to the exact nature of this disease; but still if previous to tubercular development, we can diagnosticate these alterations in the blood, the mortality of this disease will be very materially lessened; we may hope in almost every instance to prevent the deposit from taking place. And what are the symptoms of this condition? I have spoken of the changes in the mucous membranes. This is one of the most constant symptoms; morbid sensitiveness of the mucous membrane as indicated by the frequent occurrence of diarrhœa, broncorrhœa, bronchitis, &c.; such persons are liable upon the slightest exposure to contract *cold*, and this often of the most obstinate character, lasting for several weeks or months. Diarrhœa though not so frequent or so obstinate in this stage, is often present: in the more advanced stages it becomes one of the most prominent and threatening symptoms, when it is referred to tubercular enteritis; but in the post-mortem examinations of subjects when diarrhœa has been obstinate, there is often an entire absence of tubercular matter. Females are also liable to most exhausting leucorrhœa. This may or may not be accompanied by suppression of catamenia. Hæmoptysis is also a symptom of this stage, and by far the most important symptom; depending directly upon those alterations in the blood which have been mentioned as constituting the primary disease. It also occurs in the advanced stages as an effect of the tubercular deposit, and hence pathologists are accustomed to refer it, in all cases, to the presence of these foreign bodies. But how can tubercles cause hæmoptysis? They may be deposited in the course of a blood-vessel, compressing or entirely obliterating it and causing congestion, or they may excite inflammation in the parts and an afflux or congestion takes place as a consequence of this inflammation. Hæmoptysis may then occur as an effort of nature to remove the superfluous blood in the part. In the still more advanced stages this inflammation terminates in ulceration, which extending to the large vessels occasionally gives rise to the most alarming and fatal hemorrhages; but this last cause commonly operates in the more advanced stages of phthisis, and cannot be referred to in explanation of that profuse hemorrhage which occurs months or years before the slightest tubercular deposit can be detected by the nicest physical examination. Neither can congestion be the morbid condition upon which this depends, for whenever hemorrhage is an effort of nature to relieve a congested organ, so soon as that object is accomplished the hemorrhage ceases. Now from the conformation of the lungs at the summit where tubercles are almost uniformly deposited first, an additional half ounce of blood could not exist without causing such alterations, both in the respiration and in the density of the lungs, as to be detected by auscultation and percussion. Hæmoptysis in the earliest stage of phthisis before any local disease is appreciable, often occurs to the extent of ten, fifteen, or thirty ounces in the course of a few hours; it cannot be that this is merely to relieve congestion, for that degree of congestion would almost entirely obliterate, not only the vesicular but the tubular portion of the lungs. What then is the cause of hæmoptysis in this stage? Andral states that the hemorrhagic diathesis consists in a diminution of the fibrin of the blood. To this alteration, therefore, together with an increase in the serous or watery portion, and a diminution in the globules, may be referred the strong tendency to hæmomtysis manifested in this stage. But you may ask why, if this hemorrhage depends upon an alteration of the blood, does it occur from one organ rather than another? Why are the lungs almost exclusively the seat of hemorrhage? No explanation so far as I am aware, has ever been attempted, and yet a series of experiments performed by Professor Horner, without reference to this subject, offered a very plausible, if not a perfectly satisfactory explanation of this selection. Professor H. discovered a direct communication existing between the bloodvessels of the lungs and bronchial tubes, so that water poured into these bloodvessels or into the cavities of the heart, passed freely into the bronchial tubes. These experiments were made upon the lungs of man as well as of inferior animals, and the result was the same in all. Now supposing this delicate capillary communication to exist; while the blood is healthy, possessing its natural thickness and cohesiveness, it does not pass out of its ordinary course, but when it becomes thinner and looses this cohesiveness, it then passes freely from the vessels, through any outlet however small which may present itself into the bronchial tubes and is coughed or spit up.

Transparency or brilliancy of the eye is another symptom common to this stage. These symptoms, occurring in persons strongly predisposed to phthisis, either heriditarily or from conformation of chest, age, or any other cause whatever, indicate the actual existence of the disease, and in that stage when a prermanent cure may be effected.

Louisville, 1845.

